# EP34 Ovarian cancer presenting as primary Sjögren’s syndrome: a rare but important mimic

**DOI:** 10.1093/rap/rkaa052.033

**Published:** 2020-11-03

**Authors:** Saadia Sasha Ali, Arti Mahto

**Affiliations:** King's College Hospital, London, United Kingdom

## Abstract

**Case report - Introduction:**

Sjögren’s Syndrome (SS) is a chronic autoimmune inflammatory condition characterized by lymphocytic infiltration of the lacrimal and salivary glands resulting in dry eyes and mouth. One third of patients present with systemic extra glandular manifestations, including neurological symptoms. Sjögren’s syndrome as a paraneoplastic mimic to ovarian cancer has been rarely reported in the literature. We present the case of a 49-year-old female patient who was referred to rheumatology with features suggestive of Sjögren’s syndrome which were likely to have been triggered by an underlying primary ovarian malignancy.

**Case report - Case description:**

A 50-year-old female was referred from neurology with a three-week history of right sided facial numbness affecting her lower lip and chin, fatigue, and concomitant oral sicca syndrome. She also experienced facial allodynia to the right side of her lower lip. There was no associated fever, night sweats or weight loss.

Her past medical history was unremarkable except for mild eczema. Physical examination revealed marked reduction to soft and sharp touch with normal two-point discrimination. Dryness of the mucosa was noted on examination of the oropharynx. The remainder of her neurological examination was unremarkable. Her cardiovascular, respiratory, and abdominal examination revealed no abnormality. There was no enlargement of the salivary glands, cervical adenopathies, joint pathology or rashes.

Her erythrocyte sedimentation rate and C-reactive protein were elevated at 53mm/hr (0-22mm/hr) and 27mg/L (0-5mg/L) respectively. Immunology revealed a positive Ro-52 antibody on the extended ENA panel but negative ANA. Her full blood count, renal and liver function, immunoglobulins and protein electrophoresis, haematinics and HBA1c were within normal limits. Schirmer's testing was also negative. Magnetic resonance imaging of the brain showed bilateral cisternal trigeminal nerve pathological enhancement with extensions into the deep divisions with her face, raising the possibility of a vasculitic process. Despite her age, sex, xerostomia, and presence of Ro-52 antibodies which may be suggestive of primary Sjögren’s syndrome, she did not meet the 2016 classification criteria. Considering these findings, raised inflammatory markers and equivocal antibodies, she underwent an FDG PET scan which showed the presence of a primary ovarian malignancy with metastatic spread to her mediastinal lymph nodes and peritoneal tumour deposition. The patient has subsequently been referred to the gynae-oncology team for further grading, staging and consideration of chemotherapy.

**Case report - Discussion:**

There is a well-established association between Sjögren’s syndrome and haematological malignancy, notably non-Hodgkin’s lymphoma. It is also known that paraneoplastic autoimmune rheumatic syndromes such as the idiopathic inflammatory myopathies can precede the clinical manifestations of solid organ tumours. Sjögren’s syndrome as a paraneoplastic mimic to ovarian cancer has been rarely reported. One cohort study of 111 patients investigating the incidence of non-lymphoid cancers in SS documented only a sole case of ovarian malignancy.

Sensory trigeminal neuropathy in association with Sjögren’s ’s syndrome has been reported and is characterised by numbness and hyperaesthesia to the face. The prevalence of this presentation varies, but one large case series reported that 17% (15/92) had a pure sensory trigeminal neuropathy, six had symmetrical involvement. While the distribution and character of this patient’s neuropathy could be explained by a Sjögren’s related sensory syndrome, there is overlap in the underlying pathogenesis in the development of Sjogren’s associated polyneuropathy and paraneoplastic neurologic syndromes (PNS).

In SS an autoimmune vasculitic process and autoantibodies are thought to be contributors to the pathogenesis of nerve damage. There is a suggestion that trigeminal neuropathy occurs secondary to vasculitis or ganglionitis. Similarly, autoimmune processes are implicated in pathogenesis of PNS where the driving hypothesis is that tumours express antigens present on nervous system tissues. Several paraneoplastic antigens have been described, including Ro-52 antibodies. Of interest, one study reported the co-existence of Ro-52 and Jo-1 antibodies in patients with anti-synthetase syndrome appeared to confer a higher risk of malignancy and a further small study of 38 Ro-52 positive patients reported that 8 (18%) had past or present malignancy.

We felt that the acute onset of this patient’s sicca symptoms and trigeminal nerve enhancement on MRI scan warranted further investigation for a more sinister underlying pathology.

**Case report - Key learning points**
 

1. Ovarian cancer can present as a mimic of Sjögren’s syndrome
2. Up to one third of patients with primary Sjögren’s syndrome can present with extra-glandular manifestations, including cranial nerve neuropathies. Sensory trigeminal neuropathies have been reported in the literature and can occur bilaterally
3. The rapid onset of symptoms and the absence of other classification criteria should prompt further investigations to characterise the disease further
4. Ro-52 antibodies have been associated with Sjogren’s syndrome and systemic lupus erythematosus, but also in some small studies with an increased risk of malignancy. Further studies are warranted into the significance of isolated Ro-52 positivity.
5. Is it important for rheumatologists to remain vigilant for co-existing malignancies, particularly breast and ovarian cancer?
6. Finally, this was a completely unexpected diagnosis for our patient who had seen two different specialities prior to a rheumatological assessment and has now been referred to the gynae-oncology specialist nurse for further support. Empathy, effective communication, and multi-disciplinary team working remain pivotal to our speciality.

